# Unraveling the impact of water deficit stress on nutritional quality and defense response of tomato genotypes

**DOI:** 10.3389/fpls.2024.1403895

**Published:** 2024-06-18

**Authors:** Ayesha Wadood, Amjad Hameed, Saba Akram, Maria Ghaffar

**Affiliations:** ^1^ Plant Breeding and Genetics Division, Nuclear Institute for Agriculture and Biology (NIAB), Faisalabad, Pakistan; ^2^ Nuclear Institute for Agriculture and Biology College, Pakistan Institute of Engineering and Applied Sciences (NIAB-C, PIEAS), Faisalabad, Pakistan

**Keywords:** water deficit stress, tomato, MGIDI, nutrition, biochemical, physiological

## Abstract

Water deficit stress triggers various physiological and biochemical changes in plants, substantially affecting both overall plant defense response and thus nutritional quality of tomatoes. The aim of this study was to assess the antioxidant defense response and nutritional quality of different tomato genotypes under water deficit stress. In this study, six tomato genotypes were used and subjected to water deficit stress by withholding water for eight days under glass house conditions. Various physiological parameters from leaves and biochemical parameters from tomato fruits were measured to check the effect of antioxidant defense response and nutritional value. Multi-trait genotype-ideotype distance index (MGIDI) was used for the selection of genotypes with improved defense response and nutritional value under water deficit stress condition. Results indicated that all physiological parameters declined under stress conditions compared to the control. Notably, NBH-362 demonstrated resilience to water deficit stress, improving both defense response and nutritional quality which is evident by an increase in proline (16.91%), reducing sugars (20.15%), total flavonoids (10.43%), superoxide dismutase (24.65%), peroxidase (14.7%), and total antioxidant capacity (29.9%), along with a decrease in total oxidant status (4.38%) under stress condition. Overall, the findings suggest that exposure to water deficit stress has the potential to enhance the nutritional quality of tomatoes. However, the degree of this enhancement is contingent upon the distinct genetic characteristics of various tomato genotypes. Furthermore, the promising genotype (NBH-362) identified in this study holds potential for future utilization in breeding programs.

## 1 Introduction

Abiotic stresses drastically impact crop productivity globally, resulting in substantial yield losses. Global warming poses a serious threat to agriculture as a result of declining food productivity and quality, predominantly due to extreme temperatures and water deficiency (Pachauri et al., 2014; Sehgal et al., 2018). Plants are capable of adapting to environmental conditions; however, natural processes such as adaptation and the increase of genetic variability cannot keep up with rapid climate change (Dhankher and Foyer, 2018). To tackle this concern, there has been a substantial surge in the volume of scientific literature published between 2002 and 2016. This literature predominantly explores abiotic stress factors, plant reactions, and the identification of species that exhibit resistance or tolerance while maintaining high yields and nutritional value (Giordano et al., 2021).

Globally, tomatoes (*Solanum lycopersicum*) are consumed as fresh vegetables because they contain high levels of essential nutrients, antioxidants and phytochemicals. Tomato fruit contains proteins including enzymes, vitamins, sugars, monounsaturated fatty acids (linoleic and linolenic acids), amino acids, phenolics, carotenoids and flavonoids (Ali et al., 2020).

Exposure to either water or osmotic stress triggers the formation of reactive oxygen species (ROS), which can be highly detrimental to plant cells, leading to oxidative damage and the deactivation of essential enzymes. In response to this threat, cells initiate a defense mechanism by producing antioxidants that scavenge ROS, as highlighted by (Atkinson et al., 2011). A variety of strategies have evolved in plants to combat oxidative stress, including the synthesis of antioxidants and activation of stress response pathways (Mishra et al., 2023). Notable antioxidant enzymes found in plants encompass, ascorbate peroxidase (APX), superoxide dismutase (SOD), catalase (CAT), dehydroascorbate reductase (DHAR), monodehydroascorbate reductase (MDHAR), glutathione peroxidase (GPX), glutathione S-transferase (GST) and glutathione reductase (GR). Additionally, non-enzymatic antioxidants such as tocopherols, ascorbate, carotenoids, glutathione and flavonoids, as reported by (Klunklin and Savage, 2017; Sun et al., 2020), play a pivotal role in safeguarding plant cells against oxidative stress. However, the amount of these metabolites varies in different genotypes that reflect their degree of resilience and nutritional quality. The potential of food to accumulate bioactive health-promoting compounds is a crucial determinant of its functional quality (Martí et al., 2018). Tomatoes and their derivatives are rich in carotenoids, such as lycopene, ascorbic acid (AsA), and phenolic compounds, which contribute to their nutritional value, color, and flavor. The composition of tomatoes depends on genetics, ripeness, and ecological conditions (Borguini and Ferraz Da Silva Torres, 2009; Shah et al., 2015). Carotenoids, particularly lycopene, exhibit potent antioxidant properties and have been associated with a reduced risk of certain cancers (Pék et al., 2014). Tomatoes also contain phenolic compounds and flavonoids, which are abundant in plants and have therapeutic potential in various diseases (Pavlović et al., 2017). However, different tomato genotypes and environmental conditions affect tomato nutritional composition.

Water deficit stress is a major abiotic stress that negatively impacts crop growth and productivity worldwide by inducing physiological and biochemical changes that affect plant growth, development, and yield. However, tomato fruit quality may be improved by abiotic stress due to increased levels of soluble solids (sugars, amino acids, and organic acids), which accumulate inside the fruit (Nuruddin et al., 2003; Yin et al., 2010). Since soluble solids affect the flavor, taste, and water content of fresh fruits, a rise in their soluble solids increases their value. Water deficit stress-tolerant crops with enhanced nutritional value are gaining interest due to global population growth and water scarcity (Sharma et al., 2021).

Recent studies suggest that tomato may exhibit positive responses to water deficit stress, resulting in improved nutritional quality (Conti et al., 2022; Dere et al., 2022). Water deficit stress can enhance the concentration of secondary metabolites, such as phenolic compounds, flavonoids, and carotenoids, in tomato fruit, thereby improving human health (Yadav et al., 2021; Dere et al., 2022). These compounds possess antioxidant properties, which scavenge reactive oxygen species and prevent oxidative damage to cells, and improve the nutritional quality of tomato fruit by increasing the concentration of essential micronutrients, such as vitamins C and E, and minerals like potassium and calcium (Conti et al., 2022).

In this view, present study was conducted to evaluate the drought tolerance of tomato genotypes using physiological traits. Other objective was to check the effect(s) of water deficit stress on nutritional quality and defense response of tomato genotypes.

## 2 Materials and methods

### 2.1 Experimental design and water deficit stress treatment

**Figure 1:**
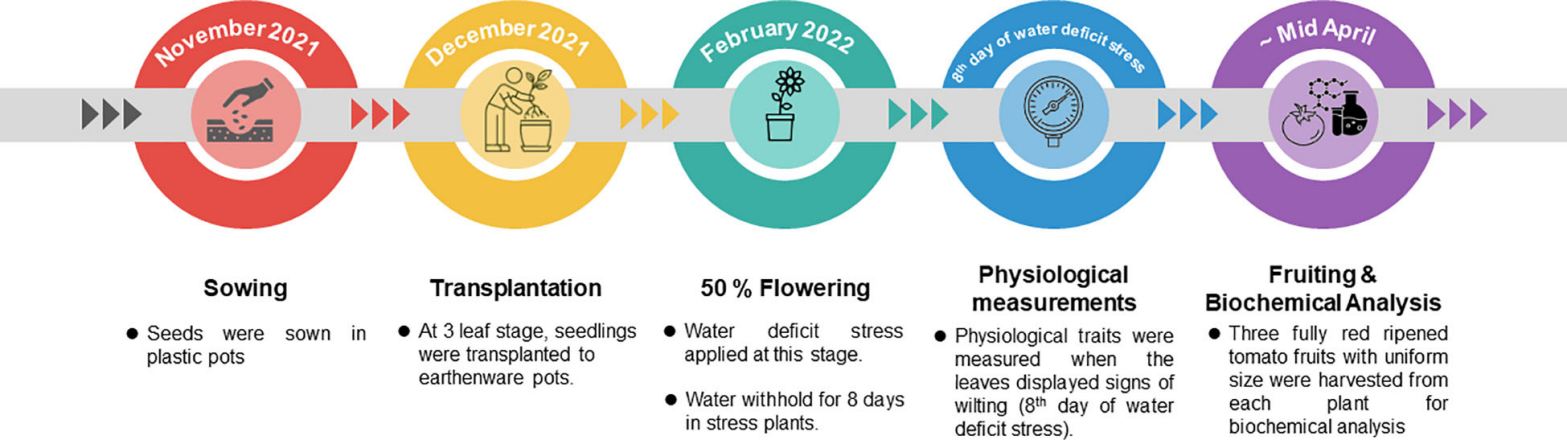
Timeline of experiments under study.

The experiment was structured as complete randomized design (CRD) with three replications in glass house at Nuclear Institute for Agriculture and Biology (NIAB). Six tomato genotypes were used (**Table 1**). Seeds were sown in plastic pots in November 2021. Seedlings were then transplanted in earthenware pots (13 inches in height and 10.5 inches in width) at three leaf stage in December 2021. Each pot was filled with air-dried and sieved soil. Fertilizers (1.1g of N (Urea), 1.1g of P (DAP) and 1.1g of K (SOP)) were at the time of transplantation, then at flowering stage and then after first fruit picking. After the application of fertilizers, watering was administered regularly until the flowering stage. At the onset of flowering, the watering regimen diverged: control plants received regular watering, while water was withheld for stress plants for a duration of 8 days to induce stress. This strategy aligns with the approach outlined in Rawal et al. (2022). Following this stress period, all plants resumed regular watering. These tests aimed to evaluate the impact of the applied stress on fruit characteristics (**Figure 1**).

**Table 1:** Detailed description of genotypes.

Sr. No.	Genotypes	Pedigree
1.	NIAB Johar	NB-13 × NB-6
2.	NIAB Gohar	NB-4 x NB-266
3.	NBH-173	NB-327 x NB-285
4.	NBH-362	NB-333 x NB-11D
5.	NIAB Tomato-21	NB-242 x NB-327
6.	NB-187	V-19905

### 2.2 Evaluation of physiological traits

Different physiological traits were measured when the leaves displayed signs of wilting (8^th^ day of water deficit stress). Further, stomatal conductance, photosynthetic rate and transpiration rate were determined during water deficit stress condition using portable porometer (LI-COR, inc, Model: LI-1600). Relative chlorophyll contents were determined by SPAD. Relative water contents of stressed and control plants were also measured. Porometer `and SPAD values were measured from two fully expanded leaves per plant, specifically the fourth and fifth leaves. For porometer reading, measurements were performed in the morning (10:00–11:30 a.m.) under a steady photon flux density of 300 mol m^-2^ s^-1^, while leaf temperature ranged between 20-22°C.

#### 2.2.1 Stomatal conductance

Using porometer, leaf temperature (LT) and diffusible resistance (DR) of control and stressed plants were measured and then SC (mmol m^–2^ s^–1^) was measured using formula as described by (Munawar et al., 2021).


$$SC=\frac{1}{DR}\times CF$$

Where CF is a correction factor which in this case is 10.

#### 2.2.2 Transpiration rate

Similarly, transpiration rate (µgcm^–2^ s^–1^) was also determined by porometer by dividing the transpiration value obtained from porometer with 10,000 and multiplying with 1000 (Munawar et al., 2021).

#### 2.2.3 Photosynthetic rate

In the same manner, photosynthetic rate was calculated by using formula described by (Munawar et al., 2021).


$$Ph=\;\frac{\mathrm{SC}\;\left(\mathrm{mmol}\;m^{-2}s^{-1}\right)}{\mathrm{Tr}\;\left(\text{μg}\;c{\text{m}}^{-2}\;{\text{s}}^{-1}\right)}\times 10$$

#### 2.2.4 Determination of relative chlorophyll contents

After eighth days of water deficit stress treatment, relative chlorophyll contents were measured using a portable device called SPAD on the top leaves of both stress and control plants. The device immediately measured RCC by simply clamping the meter on the leaf and obtaining the values on the scale.

#### 2.2.5 Relative water contents

Relative water contents of stressed and control plant leaves were measured by using method described by (Barrs and Weatherley, 1962). Briefly, Terminal leaflets from the second leaf of each plant were taken (on 8^th^ day of water deficit stress) and their fresh weight (FW) were measured immediately. These were then immersed in distilled water in a test tube and were placed in the dark for 24hrs. After that the leaflets were pat dried with tissue paper without damaging and measured their turgid weight (TW). Further these leaflets were wrapped in brown envelopes and incubate at 70°C in oven for 24 hrs. for complete drying. The dried leaflets were reweighed and obtain their dry weight (DW). RWC were then measured using formula:


$$RWC\;\left(\%\right)=\left(\frac{FW-DW}{TW-DW}\right)\;\times 100$$

### 2.3 Biochemical parameters

To investigate the water deficit stress induced biochemical changes, different biochemical parameters were measured. All these experiments were carried out in Marker Assisted Breeding (MAB) Lab I, Nuclear Institute for Agriculture and Biology (NIAB), Faisalabad. Three tomato fruits with uniform size were harvested from each plant upon reaching full ripeness, characterized by a red coloration (visual observation), typically occurring around 100 to 120 days after transplantation and stored at -20°C for later use for biochemical analysis. Fruit samples were consistently harvested promptly upon reaching full red ripeness to ensure accurate analysis.

#### 2.3.1 Ascorbate peroxidase activity

0.2 g of tomatoes at the ripening stage of each plant were extracted with 2 ml of potassium phosphate buffer (50 mM). The pH of the buffer was adjusted to 7.4. After extraction, samples were centrifuged at 4°C and 15,000 rpm for approximately 10 minutes. The supernatant containing soluble proteins was collected from each sample and used for further biochemical testing using method (Khalid and Hameed, 2017). Data were collected in triplicate. To determine enzymatic activity, we measured the rate of H_2_O_2_-mediated ascorbic acid oxidation in a reaction mixture containing 200 mM potassium phosphate buffer, pH 7, 10 mM ascorbic acid, 0.5 M EDTA, and 50 μL enzyme extract. Ascorbic acid oxidation was measured by monitoring the decrease in absorbance at 290 nm every 30 seconds for three minutes after the addition of 1 mL of a 10 mL solution of H_2_O_2_ (Chen and Asada, 1989).

#### 2.3.2 Catalase activity

0.2g of ripened tomato fruit was homogenized in a medium containing 50 mM potassium phosphate buffer of pH 7.0 and dithiothreitol (DTT) of 1 mM concentration. A supernatant containing soluble proteins was obtained by centrifuging the mixture at 15000g for 10 minutes. An enzyme extract of 0.1 ml was prepared along with 59 mM H_2_O_2_ and 50 mM phosphate buffer (pH 7.0) to measure CAT activity. Over the course of 1-2 minutes, the absorbance at 240nm decreased every 20 seconds. A change in absorbance of 0.01 Umin-1 was considered one unit of CAT activity. By dividing the acquired Umin1 value by the quantity of protein utilized in the experiment, catalase activity was normalized. According to the weight of each fruit, CAT activity was expressed (Beers and Sizer, 1952).

#### 2.3.3 Peroxidase activity

A solution containing potassium phosphate buffer (50 mM, pH 7.0), EDTA (0.1 M), and DTT (mM) was used to homogenize about 0.2 g of fruit. The supernatant from the centrifugation of the homogenate at 15000 g for 20 min at 4°C served as the enzyme extract. A test solution was made by mixing 545 ml of distilled water with 15 l of the enzyme extract, 200 1 mM of guaiacol, 400 mM of H_2_O_2_, 200 mM of phosphate buffer (pH 7.0) and 200 mM of guaiacol. The reaction was started once the enzyme extract was added, and the rise in absorbance at 470 nm was measured every 20 seconds for two minutes. One unit of POD activity was considered an absorbance change of 0.01 Umin^-1^ (Hameed et al., 2014).

#### 2.3.4 Superoxide dismutase activity

For the estimation of SOD activity, 0.2 g of fruit samples were homogenized in an extraction containing potassium phosphate buffer (50 mM, pH 7.0), EDTA (0.1 mM), and DTT (1 mM) (Hameed et al., 2014). The ability of SOD to prevent the photochemical reduction of nitroblue tetrazolium (NBT) was used to measure its activity. One unit of SOD activity equals 50% inhibition of NBT photochemical reduction (Giannopolitis and Ries, 1977).

#### 2.3.5 Total antioxidant capacity

To measure TAC, 2,2′-azino-bis (3-ethylbenzothiazoline-6-sulfonic acid) (ABTS) test, which was described by (Nenadis et al., 2007) was used. For this, 1 ml of 50 mM potassium phosphate buffer (pH 7.0) was used to homogenized 0.1g of fruit. The supernatant obtained after centrifuging the homogenate at 14000 g for 10 min at 4°C was utilized as the sample extract. Due to the presence of antioxidants in the sample, 2,2-azino-bis (3 ethylbenzothiazoline-6- sulfonate) radical cation (ABTS+), which exhibits a blue-green color, is converted into its original colorless form for this assay. Sample extract, reagent 1 (0.4M sodium acetate + 0.4M glacial acetic acid), and reagent 2 (30mM sodium acetate + 30mM glacial acetic acid) are all included in the reaction mixture. The reaction mixture’s absorbance was measured at 660 nm.

#### 2.3.6 Total oxidant status

Utilizing the procedure outlined by (Erel, 2005), total oxidant status was assessed. For this test, 0.1g of fruit was homogenized in 1 ml of 50 mM potassium phosphate buffer (pH 7.0) medium. The homogenate was centrifuged at 14000g for 10 min at 4°C, and the supernatant that collected on the other end served as the sample extract. Reagent R1 (140 mM NaCl, 50 M xylenol orange, and 1.35 M glycerol) was combined with sample extract together with reagent R2 (5 mM ferrous ammonium sulphate and 10 mM o-dianisidine dihydrochloride). After 5 minutes of incubation at room temperature, a spectrophotometer was used to measure absorbance at 560 nm. A standard curve was made using hydrogen peroxide.

#### 2.3.7 Malondialdehyde contents

The level of malondialdehyde (MDA) in tomato fruit was measured to assess lipid peroxidation. This assay was done using a protocol described by (Heath and Packer, 1968). For this, 1 ml of 50 mM potassium phosphate buffer (pH 7.0) was used to homogenize 0.1g of fruit. The homogenate was centrifuged at 14000g for 10 minutes at 4°C, and the supernatant that was obtained was used as the sample extract. A 125 µl aliquot of the sample extract was mixed with 20% TCA that contained 0.05% TBA. After that, the mixture was incubated at 95°C for 30 minutes. The mixture was rapidly allowed to cool in an ice bath after incubation. An absorbance measurement at 532 nm was made following a centrifugation at 1.4462 g for 10 minutes, and the result for nonspecific absorption at 600 nm was subtracted. The attenuation coefficient used to calculate the MDA content was 155 mM^-1^cm^-1^.

#### 2.3.8 Total phenolic contents

(Ainsworth and Gillespie, 2007) described a micro-colorimetric approach that was used to calculate TPC. In a nutshell, a standard curve was created using different gallic acid concentrations, followed by the determination of the linear regression equation. A 0.2 gramme sample of ripe tomato fruit was homogenized in cooled 95% methanol in a mortar and pestle that had previously been kept at -20°C. Following homogenization, samples were incubated for 48 hours at 25°C in the dark. After incubation, samples underwent a 5-minute, 10,000-g centrifugation process. Supernatant was collected for further TPC analysis. 100 µl of the supernatant and 100 µl of the 10% (vol/vol) Folin-Ciocalteu reagent were combined. This mixture was vortexed thoroughly and then 800 µl of 700 mM Na2CO3 was added. Samples were then incubated at room temperature for 2 hrs. Absorbance was then taken at 765 nm. Phenolic contents which are equivalent to gallic acid in standard curve was measured by linear regression equation.

#### 2.3.9 Ascorbic acid

(Mahmood et al., 2020) described the 2,6-dichloroindophenol (DCIP) method for measuring ascorbic acid. A medium containing 1 ml of 50 mM potassium phosphate buffer (pH 7.0) and 0.1 g of fruit was homogenized. The supernatant obtained after centrifuging the homogenate at 14000 g for 10 min at 4°C was utilized as the sample extract. Only decreased ascorbic acid is measured using this technique. Ascorbic acid transforms DCIP into DCIPH2 in this procedure. As the absorbance at 520 nm decreased, this conversion was observed. To determine the concentration of ascorbic acid from unidentified samples, a standard curve was created utilizing a range of ascorbic acid values.

#### 2.3.10 Total flavonoid contents

Aluminum chloride colorimetry was employed to evaluate the total flavonoid concentrations as described by (Lin and Tang, 2007). A 0.2 gramme sample of ripe tomato fruit was homogenized in cooled 95% methanol in a mortar and pestle that had previously been kept at -20°C. Following homogenization, samples were incubated for 48 hours at 25°C in the dark. After incubation, samples underwent a 5-minute, 10,000-g centrifugation process. Supernatant was removed for additional TF analysis. Briefly, 50 µl of 10% aluminum chloride, 50 µl of 1M potassium acetate, and 1.4 mL of deionized water were combined with 200 l of sample extract and 1 mL of dH_2_O. The reaction mixture’s absorbance was measured using a spectrophotometer at 415 nm.

#### 2.3.11 Total reducing sugars (sugar contents)

For this sample was extracted using 50mM potassium phosphate buffer. Total reducing sugars were measured using a method described by (Miller, 1959).

#### 2.3.12 Proline contents measurement

Proline was also measured using a protocol explained by (Bates et al., 1973). Sample was extracted by weighing the fruit sample (0.1g) and adding 2 mL of 3% sulfosalicylic acid in a 10 mL centrifuge tube. The tubes were then placed in water bath at 100°C for 1 hr. Samples were then vigorously mixed for 15-20 sec. The chromophore containing toluene was separated from the aqueous phase and was later used for proline extract. A spectrophotometer was used to take the absorbance at 520nm. A standard curve of D-proline was used to estimate its concentration.

#### 2.3.13 Pigment analysis

Using the approach previously mentioned the amounts of lycopene and carotenoids were calculated (Lichtenthaler and Wellburn, 1983). Briefly, sample was extracted using 80% acetone followed by centrifugation at 10,000g for 5 min. A spectrophotometer was used to take the absorbance at 645, 663, and 480nm.

### 2.4 Statistical analysis

Statistical analyses were carried out using R software (version: 4.2.2). Before analysis, all the data were checked for the assumption of normality (Shapiro wilk test) and transformed where required. TOS and lycopene were log_10_(x) transformed. The means were then compared by two-way ANOVA followed by Tukey’s test for multiple mean comparison (p< 0.05). Despite transformation, SC, TR, APX, TPC, TF, AsA, TAC, Reducing Sugars, POD and Pro didn’t follow a normal distribution. Thus, a non-parametric factorial analysis was conducted, and the data underwent aligned rank transformation using the ARTool package in R (Wobbrock et al., 2011) and later on subjected to ANOVA test followed by Tukey’s test (p< 0.05). Genotypes were selected by Multi-trait Genotype-Ideotype Distance Index (MGIDI) using R package metan (Olivoto and Lúcio, 2020). For the reduction of dimensionality of data, principal component analysis (PCA) was carried out by employing FactoMineR package (Lê et al., 2008) in R. To identify the relationships between variables in a dataset, correlation network was plotted in R using “qgraph” package (Epskamp et al., 2012).

## 3 Results

### 3.1 Effect of water deficit stress on physiological parameters

**Figure 2:**
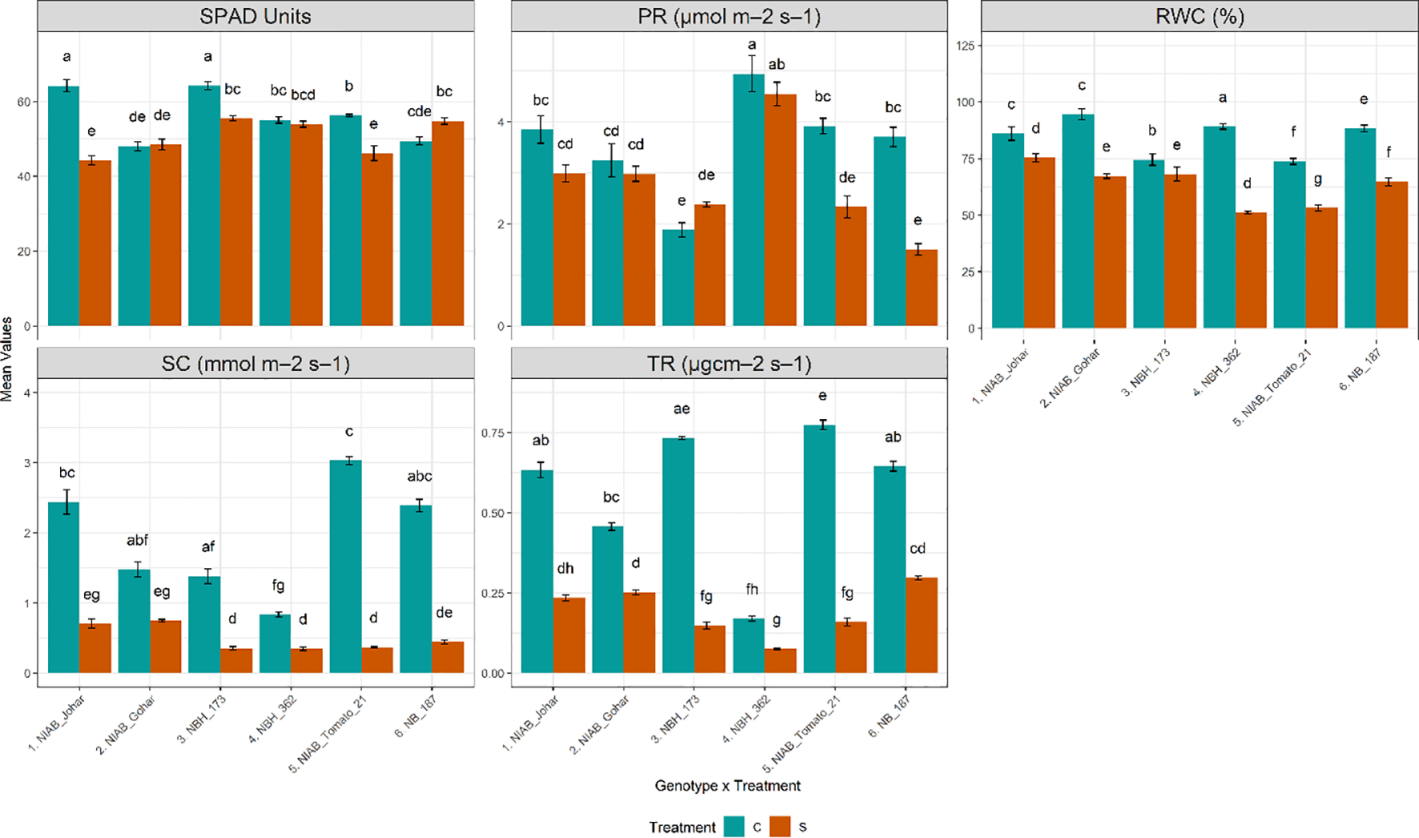
Physiological parameters of tomato genotypes under well-watered and water deficit stress conditions. The bars represent the mean data ± SE. Different letters indicate significant differences between treatments (Tukey’s *post hoc* test, p< 0.05). (SPAD value, photosynthetic rate (PR) and relative water content (RWC)) underwent Two-way ANOVA while Stomatal conductance (SC) and transpiration rate (TR) were assessed via ART Two-way ANOVA followed by Tukey’s *post hoc* test (p< 0.05). (C= control, S= water deficit stress).

The impact of water deficit stress on various physiological parameters was profound across all genotypes investigated in our study. Our observations revealed a consistent decline in almost all measured physiological parameters, including stomatal conductance (SC), transpiration rate (TR), photosynthetic rate (PR), relative water content (RWC), and SPAD values. Specifically, under water deficit stress conditions, a significant reduction in RWC was observed across all genotypes (ANOVA results provided in **Supplementary Tables S1**, **S2**), indicating a decrease in cellular water content and potential water stress-induced damage. Furthermore, the decline in SC and TR was also consistent across all genotypes, reflecting the plants’ response to water stress by reducing stomatal opening and transpirational water loss. While the majority of genotypes exhibited a significant decrease in PR under water deficit stress conditions, this reduction was particularly pronounced in NIAB Tomato-21 and NB-187. Similarly, SPAD values, indicative of chlorophyll content and leaf greenness, were significantly reduced in several genotypes under water deficit stress, including NIAB Johar, NBH-173, and NIAB Tomato-21 (**Figure 2**).

### 3.2 Effect of water deficit stress on non-enzymatic antioxidants and some other biochemical parameters

**Figure 3:**
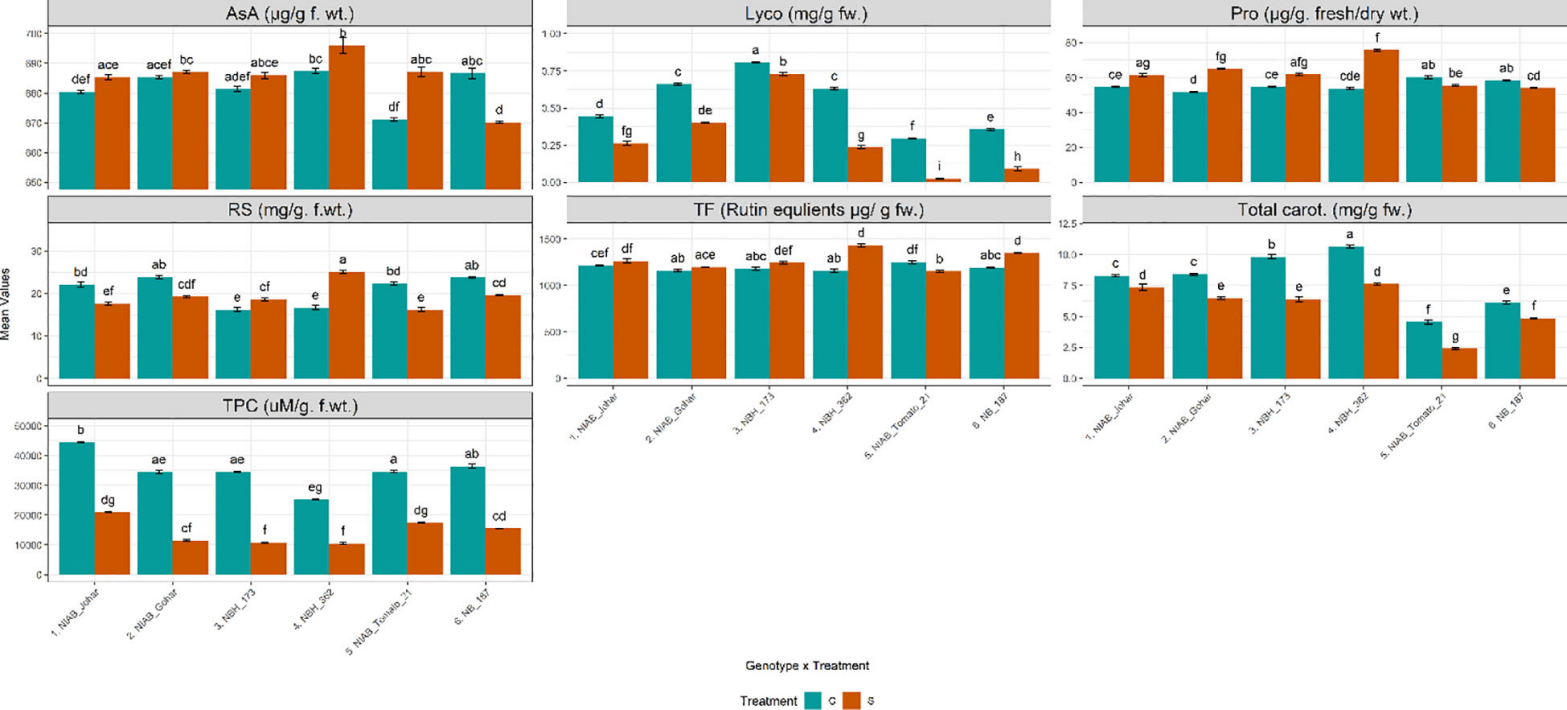
Biochemical parameters imparting direct nutritional value and defense response among different tomato genotypes under well-watered and water deficit stress conditions. The bars represent the mean data ± SE. Different letters indicate significant differences between treatments (Tukey’s *post hoc* test, p< 0.05). Ascorbic acid (AsA), proline (Pro), reducing sugars (RS), Total Flavonoid contents (TF) and total phenolic content (TPC) were assessed via ART Two-way ANOVA, while remaining parameters (lycopene (Lyco) (log_10_(x) transformed) and Total carotenoids (Total carot.)) underwent Two-way ANOVA followed by Tukey’s *post hoc* test (p< 0.05). (C= control, S= water deficit stress).

It was observed that the amount of TPC was significantly decreased under stressed condition in all genotypes (**Supplementary Tables S1**, **S2**). Another non-enzymatic antioxidant was AsA. It was observed that the amount of AsA was significantly increased only in NIAB Tomato-21 while, significantly decreased in NB-187. Total flavonoid contents (TF) which is another non- enzymatic antioxidant were also quantified. The results of TF revealed that their amount was significantly augmented in NBH-173, NBH-362 and NB-187. The amount of reducing sugars were also assessed under water deficit stress. It was observed that the quantity of reducing sugars was significantly decreased in NIAB Johar, NIAB Gohar, NIAB Tomato-21 and NB-187 under water deficit stress as compared with non-stressed control. However, this amount was significantly increased in NBH-173 and NBH-362. Proline contents in tomato fruit were also influenced by water deficit stress. It was noticed that the genotypes NIAB Johar, NIAB Gohar, NBH-173 and NBH-362 exhibited significantly higher proline contents under stress condition as compared to control. Highest proline contents were observed in NBH-362. Nonetheless, NB-187 showed significant decrease in proline contents. Lycopene and total carotenoids revealed that both are extensively affected by water deficit stress. Water deficit stress significantly reduced the amount of pigments in all genotypes (**Figure 3**).

### 3.3 Effect of water deficit stress on enzymatic antioxidants

**Figure 4:**
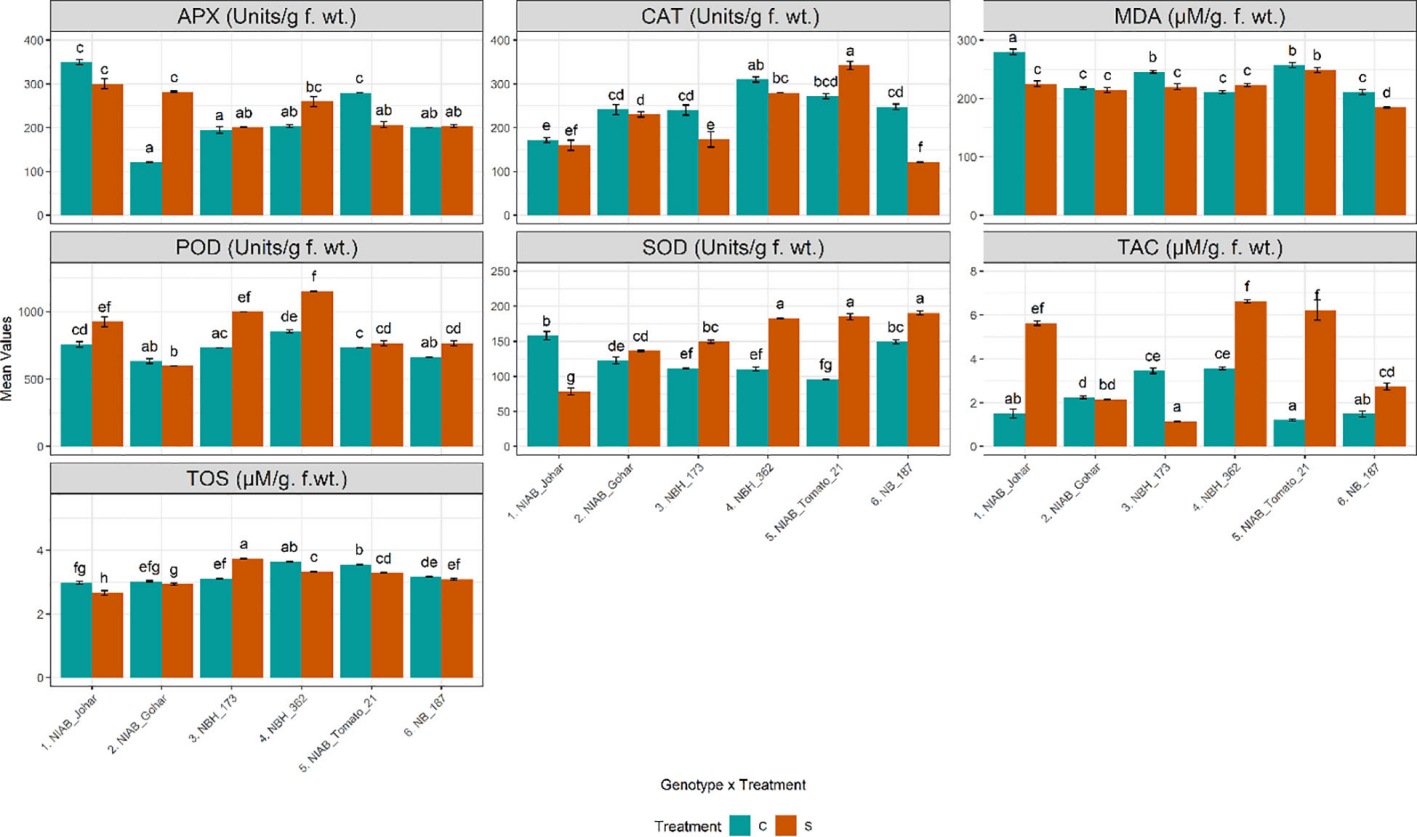
Biochemical parameters imparting defense response and indirect nutritional value among different tomato genotypes under well-watered and water deficit stress conditions. The bars represent the mean data ± SE. Different letters indicate significant differences between treatments (Tukey’s *post hoc* test, p< 0.05). Ascorbate peroxidase (APX), peroxidase (POD), and total antioxidant capacity (TAC) were assessed via ART Two-way ANOVA, while remaining parameters (catalase (CAT), malondialdehyde (MDA), superoxide dismutase (SOD), and total oxidant status (TOS) (log10(x) transformed)) underwent Two-way ANOVA followed by Tukey’s *post hoc* test (p< 0.05). (C= control, S= water deficit stress).

Water deficit stress causes increased production of reactive oxygen species which results in the increased production of enzymatic antioxidants. In this study, enzymatic antioxidants were quantified in tomato fruit to assess their scavenging ability under water deficit stress. It was observed that the amount of APX has been significantly increased only in NIAB Gohar under stressed condition. Whereas only NIAB Tomato-21 showed a significant decline in APX activity (**Supplementary Tables S1**, **S2**). Another important enzymatic antioxidant that was quantified from tomato fruit was CAT. It was found that the amount of CAT was decreased in all genotypes in stressed condition except in NIAB Tomato-21 which showed significant increase. However, the decrease in CAT concentration was significant in NBH-173 and NB-187. POD was significantly increased in NIAB Johar, NBH-173. NBH-362 and NB-187 in water deficit stress condition. Similarly, the concentration of SOD was found to significantly increase in all genotypes in stressed condition except in NIAB Johar. As for as total oxidant status is concerned, all genotypes showed decrease in oxidant status in fruit under stress condition except NBH-173 which showed a significant increase in oxidant status. However, in all other genotypes, only NIAB Johar, NBH-362 and NIAB Tomato-21revealed a significant decrease in oxidant status. TAC was progressively affected by water deficit stress. It was observed that in most of the genotypes (NIAB Johar, NBH-362, NIAB Tomato-21 and NB-187), TAC was significantly escalated under water deficit stress. Nevertheless, NIAB Gohar and NBH-173 showed a decline in TAC in stress condition but this decrease in TAC is significant in NBH-173. MDA reflects membrane lipid peroxidation and is an indicator of degree of membrane damage. In the current study, NIAB Johar, NBH-173 and NB-187 showed significant decline in MDA contents under water deficit stress (**Figure 4**).

### 3.4 Principal component analysis

**Figure 5:**
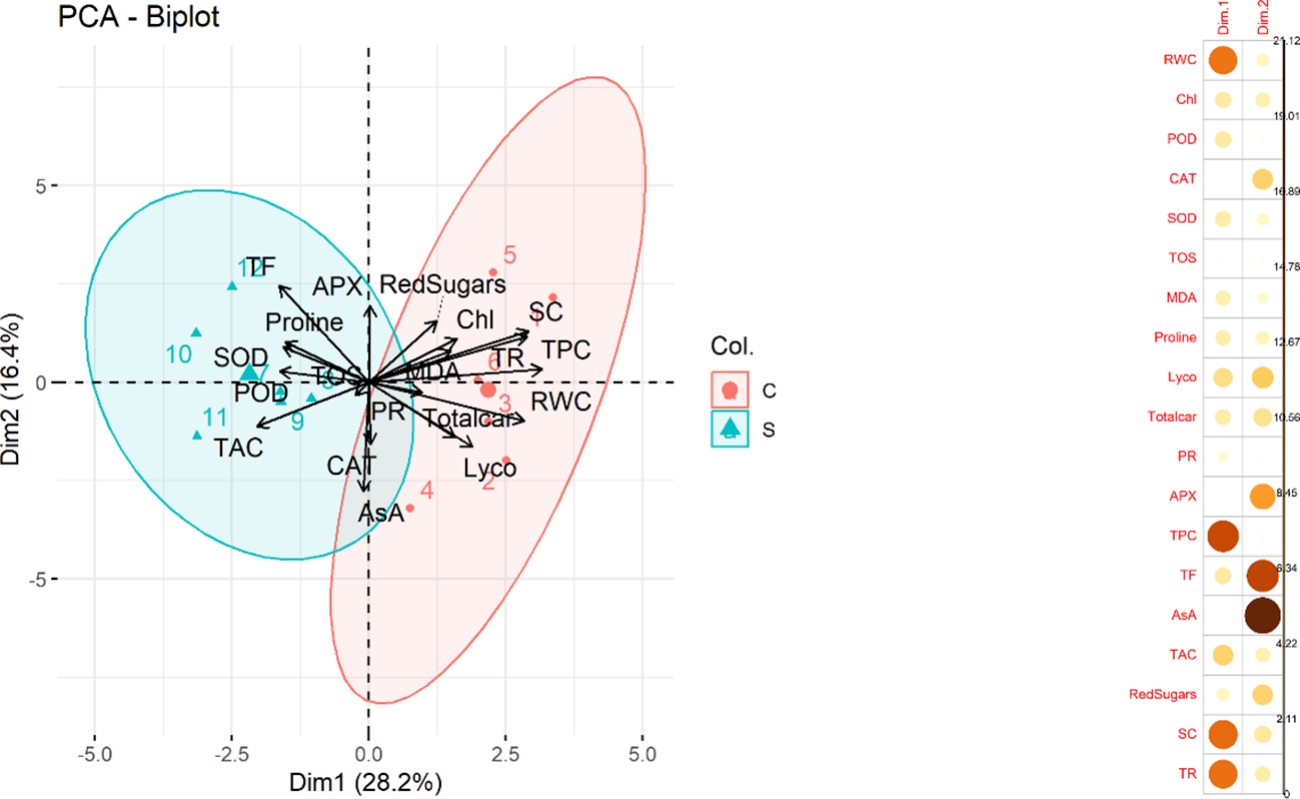
(Left) Principal Component Analysis (PCA) biplot. The plot displays the relationship between the first two principal components and the variables in the dataset. The length and direction of each arrow indicate the correlation between the variable and the principal components. The colors and shapes of the points represent the treatment (control and stress) in the dataset. (Right) Contribution of traits in PCA1 and PCA2 (Relative water content (RWC), chlorophyll content (Chl), Guaiacol peroxidase (POD), Catalase (CAT), Superoxide dismutase (SOD), total oxidant status (TOS), Malondialdehyde (MDA) content, Lycopene (Lyco), Total carotenoids (Totalcar), Photosynthetic rate (PR), Ascorbate peroxidase (APX), Total phenolic content (TPC), Total flavonoid (TF), Ascorbic acid (AsA), Total antioxidant capacity (TAC), Reducing sugars (RedSugars), Stomatal conductance (SC), and Transpiration rate (TR).

Principal component analysis (PCA) was performed to investigate the underlying structure of the dataset and to identify patterns among the variables. The results of the analysis showed that the first two principal components accounted for approximately 44.6% of the total variance in the dataset with PC1 contributing 28.2% and PC2 contributing 16.4%. A biplot (**Figure 5**) between two variables was created which explained the maximum portion of overall variation. Most contributing traits in PC1 were RWC, TPC, SC and TR. Furthermore, CAT, APX., TF, AsA and reducing sugars contributed maximally in PC2 Among these variables, CAT, APX, TF, AsA, and reducing sugars were found to be particularly relevant indicators of water deficit stress conditions.

### 3.5 Identification of the best performing genotypes through multi-trait genotype–ideotype distance index

**Figure 6:**
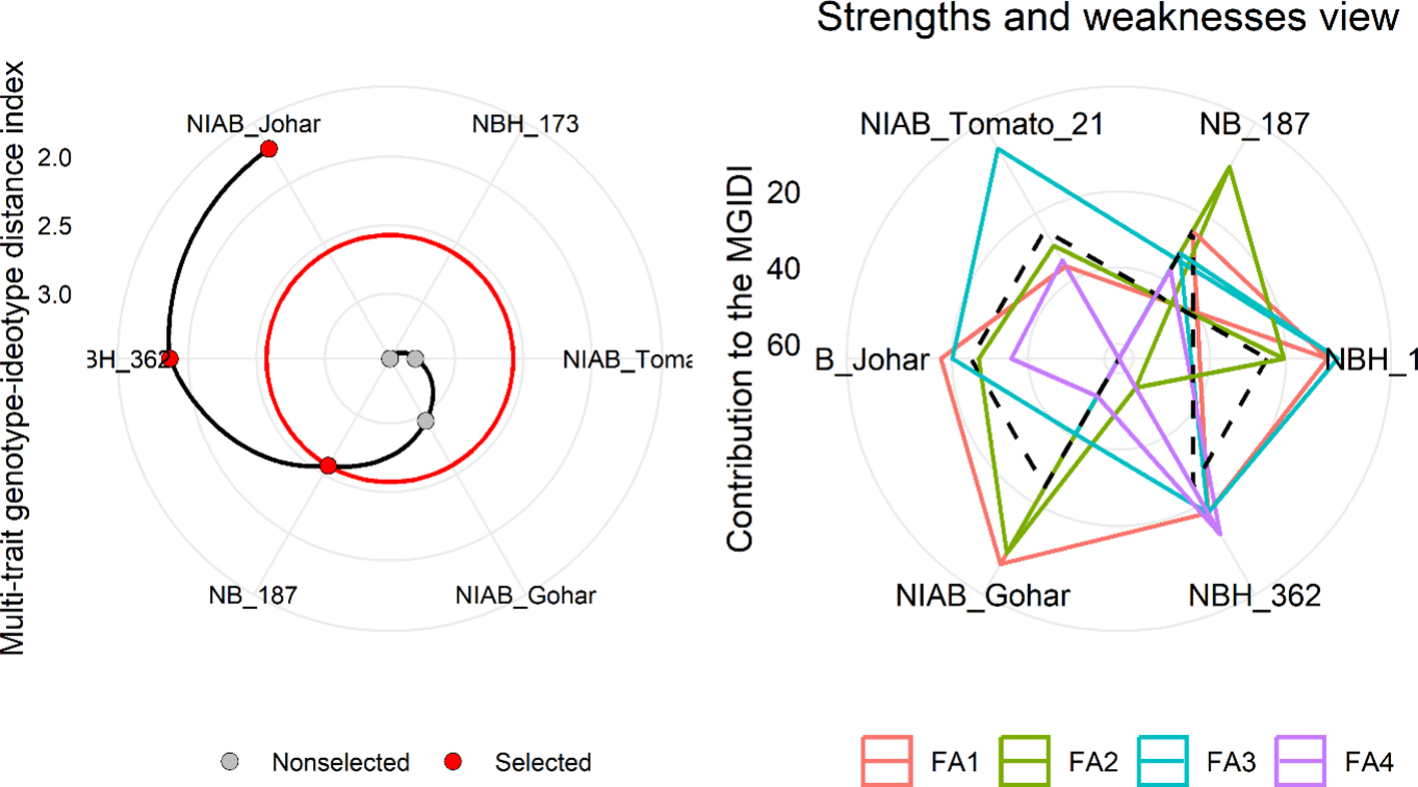
(Left) Genotype ranking under control condition using multi-trait genotype–ideotype distance (MGIDI) index. The selected genotypes based on this index are shown in red. The central red circle represents the cut-point according to the selection pressure. (right) The strengths and weakness view of treatments is shown as the proportion of each factor on the computed MGIDI. The smallest the proportion explained by a factor (closer to the external edge), the closer the traits within that factor are to the ideotype. The dashed line shows the theoretical value if all the factors had contributed equally.

**Figure 7:**
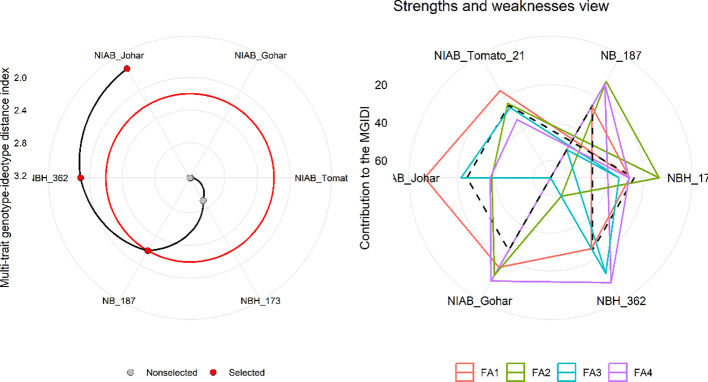
(Left) Genotype ranking under water deficit stress condition using multi-trait genotype–ideotype distance (MGIDI) index. The selected genotypes based on this index are shown in red. The central red circle represents the cut-point according to the selection pressure. (Right) The strengths and weakness view of treatments is shown as the proportion of each factor on the computed MGIDI. The smallest the proportion explained by a factor (closer to the external edge), the closer the traits within that factor are to the ideotype. The dashed line shows the theoretical value if all the factors had contributed equally.

MGIDI was used to select the best-performing genotypes under control and water deficit stress condition. This index was applied separately on control and stress condition data. The selection outputs of control as well as stress samples are presented in **Figures 6**, **7**, respectively along with strength and weakness graphs of all genotypes. According to this index, NIAB Johar, NBH-362 and NB-187 are best performing lines under controlled conditions while NBH-362, NIAB Johar and NB-187 are best performing lines in stress condition, which is equivalent to the ideotype utilized in MGIDI. However, NIAB Johar and NBH-362 are common in both control and stress condition. All filtered traits exhibited high heritability (h^2^) values. This implies that there is a promising potential for achieving selection gain in these specific traits (**Supplementary Tables S3**, **S4**).

### 3.6 Correlation

**Figure 8:**
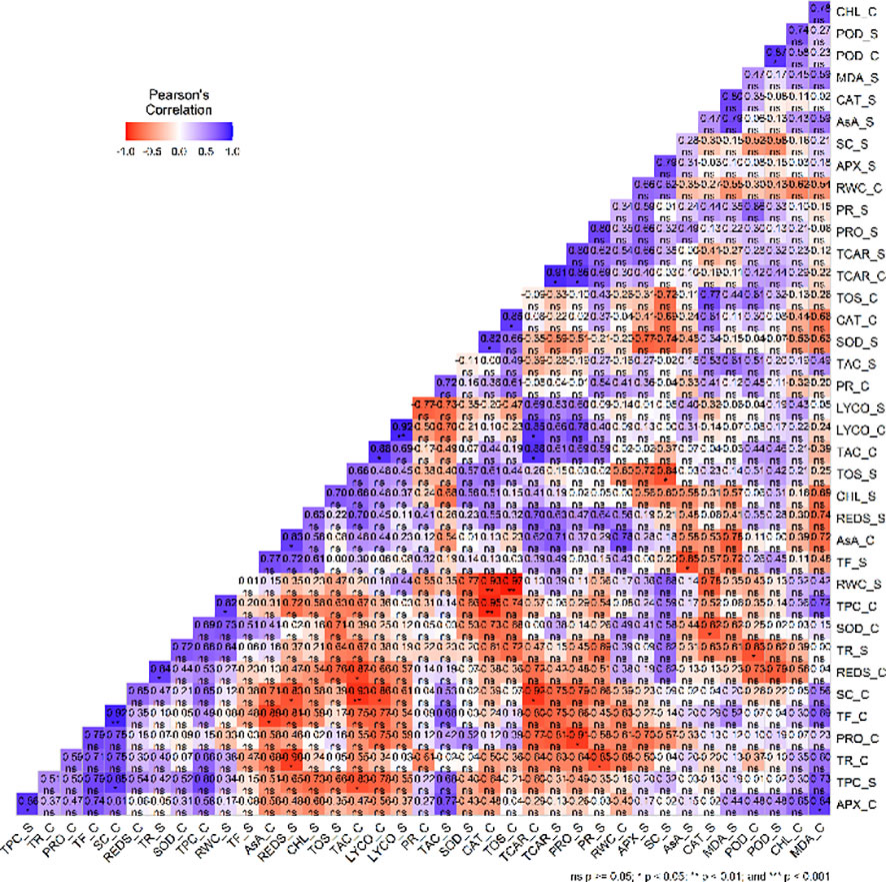
A visualization of the correlation plot of the different variables (Relative water content (RWC), Stomatal conductance (SC), Transpiration rate(TR), Photosynthetic rate (PR), chlorophyll content (CHL), Ascorbate peroxidase (APX), Catalase (CAT), Guaiacol peroxidase (POD), Superoxide dismutase (SOD), Total phenolic content (TPC), Total flavonoid (TF), Ascorbic acid (AA), total oxidant status (TOS), Total antioxidant capacity (TAC), Malondialdehyde (MDA) content, Reducing sugars (REDS), Proline (PRO), Lycopene (LYCO) and Total carotenoids (TCAR)). Blue color represent positive while red color represents negative correlation.

Correlation analysis of mean data of the different variables under control and stress conditions revealed intricate relations with different variables. In the control condition, the correlation network manifested significant associations among the variables under study (**Figure 8**). Notably, positive correlations were found between TAC and lycopene (r = 0.87*), total carotenoids and TAC (r= 0.88*) as well as SC and TF (r= 0.92**). Additionally, a positive correlation was found between MDA and APX (r= 0.84*), lycopene and total carotenoid (r= 0.86*) as well as CAT and TOS (r = 0.85*). On the other hand, negative correlations were found between SC and lycopene (r= - 0.86*), TPC and CAT (r= - 0.95**) as well as SC and both total carotenoids (r= - 0.92*) and TAC (r= - 0.93**). Additionally, a strong negative correlation was also observed between TF and AsA (r= - 0.89*) together with reducing sugars and TAC (r= - 0.8746*). In the water deficit stress condition, negative correlation was observed between SC and TOS (r= - 0.84*) as well as AsA and TF (r= - 0.85*).

## 4 Discussion

Global population growth combined with water scarcity has prompted researchers to focus on drought-resistant tomato crops that are both economically sustainable and nutritionally beneficial (Dhankher and Foyer, 2018). The intricate biochemical composition of tomato genotypes under the influence of water deficit stress is not only crucial for understanding their defense responses but also holds direct implications for the nutritional quality of these fruits, bridging the gap between plant resilience and their potential as a source of essential nutrients for human consumption (Mishra et al., 2023). In the present study, we applied water deficit stress to six tomato genotypes by withholding water for eight days and investigated the physiological parameters from leaves and biochemical parameters from fruits. Physiological traits were measured on the 8^th^ day of stress application when the leaves showed signs of wilting. At this stage, the plants were in an actively responsive state to the stress stimulus, allowing us to capture dynamic physiological changes. Conversely, the biochemical analyses assessing nutritional values were conducted on ripe fruits obtained from mature plants after the stress period. Although there is a temporal gap between physiological measurements and fruit analysis, it is important to note that our focus was on understanding the immediate physiological responses to drought stress during the critical flowering stage, which significantly influences fruit development and quality (Conti et al., 2022). By correlating physiological responses during stress with subsequent fruit quality, we aimed to elucidate the long-term impact of drought stress on plant nutritional value. Our study highlights the ability of different tomato genotypes to sustain defense mechanisms while ameliorating nutritional quality under stress, offering valuable insights for sustainable agriculture and improved fruit quality.

Decrease in RWC is one of the early symptoms of water deficiency in plant tissues (Valentovic et al., 2006). The current study suggested that RWC was decreased under water deficit stress condition in all genotypes. In line with our study, different studies showed that water shortage led to the decrease in relative water contents in tomato (Zhou et al., 2017; Patanè et al., 2022). Furthermore, it was observed that low water content adversely impacts various physiological parameters. To prevent water loss, plants tend to close their stomata which results in decline in stomatal conductance and transpiration rate. Both low water content and stomatal closure further lead to insufficient supply of CO_2_ and thus decreasing photosynthetic rate (Zhang et al., 2022). The existing data revealed a significant decrease in stomatal conductance and transpiration rate which is also suggested by (Hao et al., 2019; Ors et al., 2021). However, the photosynthetic rate was non-significantly decreased in NIAB Johar, NIAB Gohar, NBH-173 and NBH-362 that showed a degree of resilience to drought stress in these genotypes. To assess the damage caused by stress to the photosynthetic apparatus, chlorophyll content is often used as an indicator (Nankishore and Farrell, 2016). The chlorophyll content, measured as SPAD value, exhibited significant reductions in NIAB Johar, NBH-173, and NIAB Tomato-21 under drought stress conditions. In contrast, NIAB Gohar, NBH-362, and NB-187 displayed no significant impact on chlorophyll content, indicating a degree of resilience to drought stress in these genotypes.

Water deficit stress enhances the accumulation of compounds involved in antioxidant defense response, fruit taste and nutritive value by stimulating the primary and secondary metabolisms (Ripoll et al., 2014; ElSayed et al., 2019). In breeding program, fruit yield is an important parameter, but the quality of fruit can also not be overlooked. Water deficit stress compromises fruit yield but ameliorates fruit quality. However, the effect of fruit quality upon water deficit stress has seldom been investigated. To unravel different complex cascade in tomato plants upon water deficit stress, it is imperative to enhance our comprehension of how this stress affects both antioxidant defense responses and nutritional quality. In the present study, tomato plants were evaluated for drought resistance, as well as their nutritional value, through a comprehensive evaluation of enzymatic and non-enzymatic antioxidants from fruits. Among these biochemical parameters, TPC, TF, AsA, reducing sugars, proline and pigments including lycopene, and carotenoids were measured as direct indicators of tomato fruit’s nutritional value under water deficit stress. Additionally, antioxidant parameters (APX, CAT, POD, SOD, Total Oxidant Status, MDA, and Total Antioxidant Capacity) provided valuable insights into the fruit’s antioxidant potential, indirectly supporting its nutritive quality. This approach was influenced by studies showing that excess reactive oxygen species (ROS) can damage proteins, lipids, and impact fruit quality (Hariyadi and Parkin, 1991; Sala, 1998; Tian et al., 2013). By measuring antioxidant enzymes (CAT, APX, POD, SOD) and parameters like Total Oxidant Status, MDA, and Total Antioxidant Capacity, we aimed to understand the fruit’s defense against ROS-induced damage and its ability to preserve nutritional quality under water deficit stress.

Water deficit stress results in the production of a higher amount of ROS. As a result of ROS accumulation, oxidative stress occurs which damages proteins, DNA, and lipids (Gill and Tuteja, 2010). Therefore, an equilibrium is required between ROS production and their scavenging molecule (Apel and Hirt, 2004). As a defense mechanism there are different antioxidant enzymes in plants that scavenge ROS by different mechanisms. SOD initiates the first line of defense by scavenging O^2−^ in plants to form H_2_O_2_, which is then eliminated by POD, CAT and APX (Rajput et al., 2021). APX, CAT and POD scavenge H_2_O_2_ by using different mechanisms. APX depends on an ascorbate and glutathione (GSH) regeneration system while CAT directly converts H_2_O_2_ into H_2_O and 1/2 O_2_ (Sofo et al., 2015). The guaiacol peroxidase (POD) is a heme-containing protein that prefers to oxidize aromatic electron donors, such as guaiacol and pyrogallol, at the expense of hydrogen peroxide (Erofeeva, 2015). Several studies revealed escalation in the enzymatic antioxidant activity under water shortage condition (ElSayed et al., 2019; Ahmad and Li, 2021; El-Mogy et al., 2022). In the existing data, augmented APX, SOD and POD activities were observed under water deficit condition in different genotypes. However, the general trend of CAT activity was found to be decreased but it is not having a significant impact on the plants as this decrease is statistically not significant. The results of decline in CAT are also in line with (Pan et al., 2006). This could be because the plants have other mechanisms in place to protect themselves from ROS damage. In view of these results, water deficit stress increases antioxidants contributing to a robust defense mechanism and improves fruit quality.

Overproduction of ROS under stress causes lipid peroxidation and MDA accumulation, which ultimately damage cell membranes and lead to cell death. MDA is therefore considered a good indicator of membrane stability under stress (Patanè et al., 2022). Our results showed a significantly lower level of MDA contents in NIAB Johar, NBH-173 and NB-187 with respect to control. The results of MDA contents by (Patanè et al., 2022) support our current data.

Further, the amount of nonenzymatic antioxidants i.e., TPC, TF and ascorbic acid (AsA) were also measured. These antioxidants not only showed their defense response in water deficit stress but also are key components of pharmaceuticals, nutraceuticals, cosmetics, and traditional medicines (Alenazi et al., 2020; Collins et al., 2022; Cruz-Carrión et al., 2022). The amount of TPC and AsA were decreased in all genotypes under stress. In contrast to previous studies (Klunklin and Savage, 2017; Dere et al., 2022) indicating an increase in TPC and AsA levels under drought stress conditions, our findings reveal a notable decrease in these parameters within the context of tomato plants. This discrepancy could potentially be attributed to variations in experimental setups, genetic diversity among tomato cultivars, and duration of drought stress applied. Moreover, TF was increased in stress condition in all genotypes except NIAB Tomato-21. The difference in quantitative values of nonenzymatic antioxidants might be due to genotypic variation (Sarker and Oba, 2018).

Tomato is an excellent source of natural antioxidants (Cheng et al., 2017). It is therefore believed that tomatoes are beneficial due to a diverse range of antioxidative, chemoprotective, and antiproliferative activities of their dietary antioxidants (Çelik et al., 2017). In addition, these compounds decrease the impact of ROS produced during normal metabolic reactions such as cellular respiration and photosynthesis, which are caused by environmental stress or UV radiation. The ROS cause serious oxidative damage to our body’s lipids, DNA, carbohydrates, and proteins (Yang et al., 2019; Alenazi et al., 2020). The current data delineate that the amount of antioxidants are increased in water deficit stress condition conferring water deficit stress tolerance and improvement in the nutritional quality of fruit in some genotypes.

In several studies, water deficit stress has been shown to improve fruit quality by stimulating the primary and secondary metabolisms, thus enhancing the accumulation of compounds involved in fruit taste and nutrition (Ripoll et al., 2016; Medyouni et al., 2021). Osmotic adjustment is a cardinal mechanism that is adopted by plants to water deficit stress resulting in the increment of solute concentration of cells to maintain potential gradients required for the continuous uptake of water in stress condition. Moreover, osmotic adjustment ensures turgor, which is vital to plant growth and other physiological processes (Nahar and Ullah, 2017). In the present study the amount of reducing sugars and proline were also measured to examine the effect of water deficit stress response as osmotic adjustment. It was observed that the proline contents were increased in NIAB Johar, NIAB Gohar, NBH-173 and NBH-362 While sugar contents were only increased in NBH-173 and NBH-362 suggesting their response to water deficit stress. (Živanović et al., 2020) also exhibited higher proline and sugar level in tomato under water deficit condition. They explained that the accumulation of sugars and proline depends upon the abscisic acid production which is the result of stomatal closure in response to water deficit stress to avoid water loss. This might be the case in the current study too. Proline serves vital functions in protein synthesis and structure, as well as in metabolism, nutrition, wound healing, antioxidative processes, and immune responses. Requirements of proline for whole-body protein synthesis are the greatest among all amino acids. Therefore, physiological needs for proline are particularly high during the life cycle (Wu et al., 2011). Thus, genotypes with high amount of proline are good source of supplementation. Moreover, reducing sugar is a source of carbohydrate and is a part of dietary nutrient in tomato (Ali et al., 2020).

In contrast to proline and reducing sugars, lycopene and total carotenoids were decreased in stress condition. Our results are in coherence with the previous study showing the decline in pigments under water deficit stress condition in eggplant (Hannachi et al., 2022).

A recent approach, MGIDI, designed for genotype selection using multiple traits, was demonstrated by (Olivoto and Lúcio, 2020). MGIDI proves to be a powerful tool for analyzing multivariate plant data. The performance of the MGIDI index was evaluated through a Monte Carlo simulation study, comparing its success rate in selecting traits with desired gains to classical and modern indexes under various scenarios (Olivoto et al., 2022). Based on the current study’s results, different tomato genotypes exhibiting water deficit stress resilience and improved fruit quality under well water (control) and stress condition were determined by MGIDI. According to this indexing, NIAB Johar showed the overall highest quality of fruit under control condition followed by NBH-362 and NB-187 whereas, NBH-362 exhibited high nutritional value under stress condition followed by NIAB Johar and NB-187. Keeping in view these results, NBH-362 is the best genotype with water deficit stress tolerance and high nutritional value under stress conditions.

## 5 Conclusion

Taken together, NBH-362 appeared as the best genotype with notable increase in various key parameters including SOD, POD, TF reducing sugars, TAC and proline under water deficit stress as compared to control. Conversely, NIAB Johar excelled under control conditions. These findings have important implications for breeding programs and agriculture, as they identify NBH-362 as a promising genotype for drought-prone environments with high nutritive value, while NIAB Johar remains an excellent choice for optimal growing conditions.

## Data availability statement

The original contributions presented in the study are included in the article/**Supplementary Materials**. Further inquiries can be directed to the corresponding author.

## Author contributions

AW: Conceptualization, Data curation, Formal analysis, Writing – original draft, Writing – review & editing. AH: Conceptualization, Methodology, Supervision, Writing – review & editing. SA: Data curation, Writing – review & editing. MG: Formal analysis, Writing – review & editing.

## Supplementary material

Supplementary Table 1 ANOVA results.

Supplementary Table 2 Aligned rank transformation ANOVA results.

Supplementary Table 3 MGIDI_control group.

Supplementary Table 4 MGIDI_stress group.
